# The association between occupational stress level and health-related productivity loss among Korean employees

**DOI:** 10.4178/epih.e2023009

**Published:** 2022-12-28

**Authors:** Jonghee Chung, Jin-Hyo Kim, Jae Yoon Lee, Hee Seok Kang, Dong-wook Lee, Yun-Chul Hong, Mo-Yeol Kang

**Affiliations:** 1 College of Medicine, The Catholic University of Korea, Seoul, Korea; 2 Public Healthcare Center, Seoul National University Hospital, Seoul, Korea; 3 Department of Preventive Medicine, Seoul National University College of Medicine, Seoul, Korea; 4 Department of Human Systems Medicine, Seoul National University College of Medicine, Seoul, Korea; 5 Department of Occupational and Environmental Medicine, Seoul St. Mary’s Hospital, College of Medicine, The Catholic University of Korea, Seoul, Korea

**Keywords:** Occupational stress, Health-related productivity loss, Labour productivity, Work performance

## Abstract

**OBJECTIVES:**

Occupational stress management is particularly important for successful business operations, since occupational stress adversely affects workers’ health, eventually lowering their productivity. Therefore, this study aimed to investigate the correlation between occupational stress and health-related productivity loss (HRPL) among Korean workers.

**METHODS:**

In 2021, 1,078 workers participated in a web-based questionnaire survey. HRPL was measured using the Work Productivity and Activity Impairment Questionnaire, and occupational stress was measured using the Korean Occupational Stress Scale-Short Form. The occupational stress level was divided into tertiles (low, intermediate, and high), and the low occupational stress group was used as the reference group. Using a generalised linear model, differences in labour productivity loss according to the level of occupational stress were tested after adjusting for demographic characteristics such as age, gender, education level, household income, occupation, and underlying medical conditions.

**RESULTS:**

Non-parametric regression analysis of HRPL according to occupational stress showed a direct association between occupational stress and HRPL. A statistically significant difference was observed in HRPL between participants with intermediate and high occupational stress and those with low occupational stress.

**CONCLUSIONS:**

Our results support the hypothesis that high occupational stress is associated with decreased labour productivity.

## GRAPHICAL ABSTRACT

[Fig f3-epih-45-e2023009]

## INTRODUCTION

Occupational stress refers to the stress that an individual experiences during work and has the risk of negatively affecting workers’ quality of life and health status. Previous studies analysing the relationship between occupational stress and health have reported that occupational stress affects workers’ health, including cardiovascular diseases and metabolic syndrome [1], as well as health behaviours [2]. In addition, occupational stress has been shown to increase the risk of major depression [3,4], and high levels of occupational stress have been linked to an increased risk of musculoskeletal symptoms and occupational injury, particularly in emergency service organisations such as police services [5].

The consequences of occupational stress include increased labour loss, such as absenteeism and early leave [6]. It has also been established that occupational stress causes social burdens, such as a decrease in job satisfaction and engagement, and an increase in medical spending.

Hence, occupational stress management is particularly important for successful business operations since occupational stress adversely affects workers’ health, eventually lowering their productivity. In previous studies, labour productivity has mainly been measured in terms of absenteeism (being absent from work) and presenteeism (being at work but unable to show normal work performance due to health problems). The magnitude of the burden of productivity loss owing to health-related symptoms has also been reported. For instance, major depression was found to reduce worker productivity by 14.7% [7]. This pattern has been observed in Korea and other East Asian cultures such as Japan and Hong Kong [8].

In this context, understanding the correlation between workers’ occupational stress and health-related labour productivity is vital for formulating strategies to improve health and workplace productivity. Previous studies have linked occupational stress to health problems or loss of work productivity; however, few have attempted to directly correlate occupational stress with health-related productivity loss (HRPL). Therefore, this study aimed to investigate the correlation between occupational stress and HRPL among Korean workers.

## MATERIALS AND METHODS

### Study participants

We recruited 1,078 workers for this cross-sectional survey, which was conducted in 2021, and data were acquired via a web-based questionnaire. The survey included demographic information (e.g., age, gender, height, weight, and education level), economic activity, working from home, work-life balance, health behaviours, labour productivity, and occupational stress. After excluding missing values, our final sample comprised the survey results of 1,072 workers in Korea.

### Independent variable: occupational stress

The Korean Occupational Stress Scale (KOSS), which consists of 43 questions or 24 shortened questions in 8 major categories—physical environment, occupational requirements, occupational autonomy, relationship conflict, occupational insecurity, organisational structure, inappropriate compensation, and workplace culture—was developed as a tool to measure the occupational stress of Korean workers. In this study, occupational stress was measured using the Korean Occupational Stress Scale-Short Form, a self-report questionnaire consisting of 7 subdomains of work-related stress factors (job demand [Q1], insufficient job control [Q2], interpersonal conflict [Q3], job insecurity [Q4], organisational system [Q5], lack of reward [Q6], and occupational climate [Q7]) with 24 questions. Each question was rated on a 4-point Likert scale, with the lowest score being 1 and the highest being 4 [9].

The occupational stress for each of the 7 subscales was converted to a 100-point score as 100(%)× [(total response score in each item)−(number of questions in each item)]/[(highest possible questionnaire score in each item)-(number of questions in each item)]. The total score for occupational stress was obtained by averaging each item.

### Dependent variable: health-related productivity loss

HRPL was measured using the Work Productivity and Activity Impairment Questionnaire (WPAI): General Health version. The WPAI is an index consisting of 6 survey questions that reflect an overall decline in work productivity, such as absenteeism and presenteeism, among currently employed workers [10,11].

Absenteeism is defined as the degree to which a worker is absent. In this study, absenteeism was calculated as the percentage of work hours that workers were unable to attend due to health-related problems in the past 7 days. In other words, the absenteeism rate was defined as the (time missed due to health problems in the past 7 days)/[(time missed due to health problems in the past 7 days)+(time actually worked in the past 7 days)].

Presenteeism is defined as being at work but experiencing impairment while working due to health problems. Therefore, presenteeism was calculated as (effect of health problems on work productivity in the past 7 days/10) × (hours actually worked in the past 7 days)/[(hours missed due to health problems in the past 7 days)+(hours actually worked in the past 7 days)].

In this study, HRPL was defined as the extent to which workers were absent from work or too impaired to perform normally at work due to health problems over the past 7 days. Therefore, HRPL was calculated as the sum of absenteeism and presenteeism.

### Covariates

Demographic variables such as age, gender, education level, and household income were considered as covariates. Age was treated as a numerical variable and gender as a categorical variable. Education level was divided into 3 categories: (1) high school graduate or lower, (2) college or university graduate, and (3) graduate school or higher. Income was also divided into 3 categories based on the monthly income distribution: (1) less than 3 million Korean won per month, (2) between 3 million and 5 million Korean won per month, and (3) over 5 million Korean won per month. Occupations were classified into 7 categories according to the Korean Standard Classification of Occupations, which is based on the International Standard Classification of Occupations adopted by the International Labour Organization, as follows: managers, professionals, and related workers; clerks; service workers; sales workers; craft and related trade workers; equipment, machine operation, and assembly workers; and elementary workers.

### Statistical analysis

First, the participants’ demographic characteristics were identified and reported; then, the participants were divided into groups according to their demographic characteristics, and occupational stress was recorded. Second, non-parametric regression of occupational stress and health-related labour productivity loss was performed using a smoothing spline curve and generalised additive model. The relationship between the occupational stress level and HRPL was examined using a generalised linear model. The occupational stress level was divided into tertiles (low, intermediate, and high) according to its distribution and was treated as a categorical variable. The low occupational stress group was used as the reference group. Using this method, the statistical significance of differences in labour productivity loss according to occupational stress levels was tested. The regression model was adjusted for age, gender, education level, household income, occupation, and underlying medical conditions as covariates. In addition, to understand which subdomain of occupational stress most strongly affected HRPL, a generalised linear regression analysis was used to identify correlations between each subdomain of occupational stress and HRPL. All statistical analyses were performed using R version 4.2.0 (R Foundation for Statistical Computing, Vienna, Austria), and 2-tailed p-values < 0.05 were adopted as the criterion for statistical significance.

### Ethics statement

All participants signed a consent form, and anonymity and confidentiality were ensured. The study protocol was approved by the Institutional Review Board of the Seoul National University College of Medicine (C2107-253-1242).

## RESULTS

Table 1 shows the demographic characteristics of the study participants and occupational stress levels of each demographic subgroup. The total number of participants was 1,072, of whom 503 (46.9%) were men and 569 (53.1%) were women. The mean occupational stress level for the entire group was 46.63, and the standard deviation was 12.86. Women showed higher occupational stress levels on average than men. Furthermore, higher education levels and household income were associated with lower average occupational stress levels. In particular, the mean occupational stress level was higher among participants with an education level lower than high school than in those with a graduate-school-level education. The occupational stress level was the lowest among managers, professionals, and related workers, while it was the highest among sales workers. However, there were no clear trends according to age or household income.

**Figure 1.**
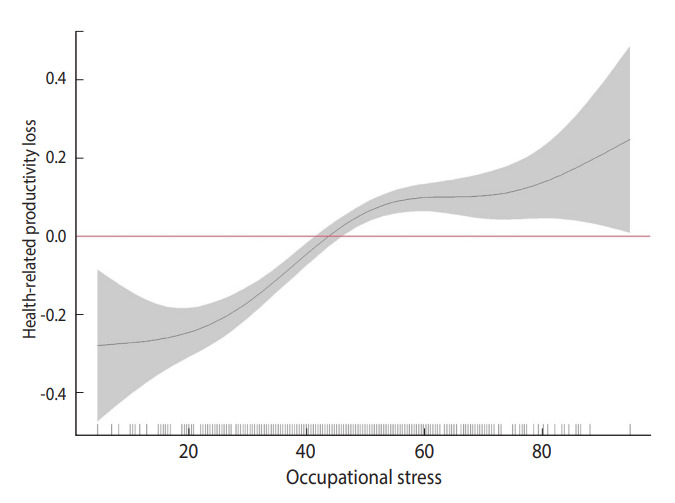
Non-parametric associations between occupational stress and health-related productivity loss.

Figure 1 shows the results of non-parametric regression analysis of HRPL according to occupational stress. The graph shows that occupational stress was directly associated with HRPL. The slope was particularly steep when the occupational stress score was between 20 and 50.

Table 2 shows the average HRPL according to the 3 levels of occupational stress (low, intermediate, and high). Three models were created and adjusted for the covariates. In all 3 models, a statistically significant difference was observed in HRPL between participants with intermediate and high occupational stress and those with low occupational stress.

**Figure 2.**
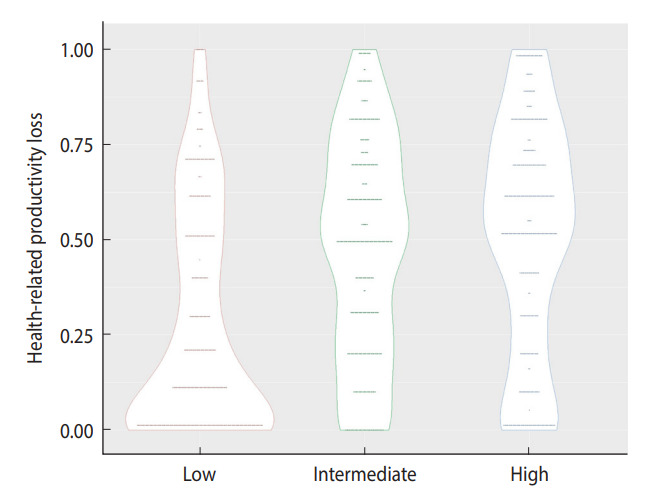
Violin plot of health-related productivity loss and its 95% confidence intervals according to the tertile of occupational stress levels.

Figure 2 shows notched violin plots displaying the distribution of HRPL according to the 3 occupational stress levels. In the plot, the HRPL distributions of the groups with intermediate and high occupational stress were different from those of the group with low occupational stress, consistent with the findings in Table 2.

Table 3 shows the average HRPL according to the subdomains of occupational stress level groups, providing a closer look at the factors that contribute to productivity loss. The groups with intermediate and high occupational stress showed statistically significant higher HRPL regarding Q1 (job demand), Q4 (job insecurity), Q5 (organisational system), and Q7 (occupational climate) than the group with low occupational stress. Meanwhile, only the group with high occupational stress showed higher HRPL regarding Q6 (lack of reward), whereas no statistically significant differences in HRPL were found regarding Q2 (insufficient job control) and Q3 (interpersonal conflict).

## DISCUSSION

To the best of our knowledge, this is the first study to investigate the relationship between occupational stress and health-related labour productivity in the Korean working population. A statistically significant correlation was observed between higher occupational stress and HRPL. In particular, the loss of labour productivity was clearly visible in participants with intermediate and high occupational stress compared to those with low occupational stress. These results echo those of a previous study by Brunner et al. [12] that assessed work productivity and workplace characteristics among Swiss employees. The results suggest that an increase in task-related and social job stressors increases HRPL, whereas an increase in social jobs and personal resources (measured by occupational self-efficacy) reduces this loss. This may be because the mental and physical health problems of workers caused by stress reduce their labour productivity.

Since the development of the KOSS, many studies have investigated occupational stress. A significant correlation has been reported between occupational stress and psychosocial health [1,2,13], and it is well established that occupational stress can affect workers’ psychological well-being, such as by causing depression, cardiovascular disease, and musculoskeletal symptoms [3,6]. On a different note, it has been found that worsened health conditions lead to labour productivity loss [7]. Although numerous studies have explored the relationship between occupational stress, worker health, and productivity loss, there is a lack of research directly linking occupational stress and HRPL.

In our analysis of the associations between HRPL and each of the 7 subdomains of work-related stress factors, statistically significant correlations were found regarding 5 subdomains: job demand, job insecurity, organisational system, lack of reward, and occupational climate. There was a clear dose-response relationship regarding the subdomains of job demand, job insecurity, organisational system, lack of reward, and occupational climate. Loss of labour productivity was found in groups with intermediate and high occupational stress in these subdomains, indicating that the management of job demand at an appropriate level with balanced rewards and favourable improvements in job stability and the occupational climate can improve labour productivity by reducing absenteeism and presenteeism. However, contrary to popular belief, among the 7 subdomains of work-related stress factors, insufficient job control, and interpersonal conflict showed insignificant results, requiring further investigation. A better understanding of these factors could help in the effective management of occupational stress and prevent future health-related labour productivity loss.

This study had several limitations. First, the representativeness of the sample is problematic; as data were collected through an Internet survey, the sample collection process may have been affected by selection bias. Moreover, because the data were not collected using a multi-stage stratified sampling method, the results may not represent the entire population of Korea. Second, because the data were collected based on participants’ subjective reports, the collection process may have been affected by information bias. Future studies should provide direct and quantitative work productivity measurements in the workplace. Finally, this study could not provide evidence for a causal relationship between occupational stress and HRPL because of the cross-sectional nature of its design. These limitations should be addressed in future research.

Nevertheless, this study demonstrated a link between occupational stress and health-related productivity among more than 1,000 workers in Korea. The results of this study are expected to be used as an important foundation for properly managing occupational stress and preserving health-related labour productivity in the future.

## Figures and Tables

**Figure f3-epih-45-e2023009:**
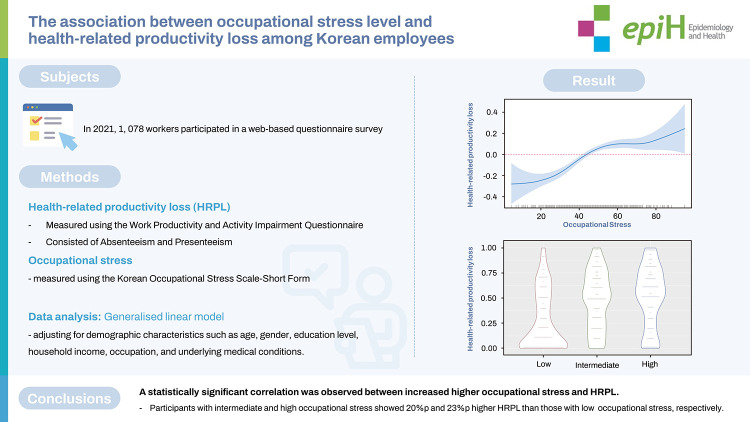


**Table 1.** Occupational stress levels according to the demographic characteristics of the study participants

Characteristics		n (%)	Mean±SD
Gender			
	Men	503 (46.9)	45.63±12.77
	Women	569 (53.1)	47.51±12.90
Age (yr)			
	20-29	197 (18.4)	46.01±12.62
	30-39	454 (42.3)	47.26±12.94
	40-49	283 (26.4)	46.86±12.80
	50-59	111 (10.3)	45.83±12.51
	≥60	27 (2.5)	41.42±14.77
Education			
	≤High school	117 (10.9)	46.29±13.19
	College or university	812 (75.7)	46.81±12.68
	Graduate school	143 (13.3)	44.35±13.43
Monthly household Income (10^4^ Korean won)			
	1st tertile (<300)	356 (33.2)	47.42±13.13
	2nd tertile (300-500)	319 (29.8)	46.60±12.22
	3rd tertile (>500)	397 (37.0)	45.93±13.11
Occupation			
	Managers, professionals and related workers	135 (12.6)	43.54±13.81
	Clerks	714 (66.6)	46.67±12.52
	Sales workers	35 (3.2)	49.61±12.35
	Service workers	90 (8.4)	46.12±13.11
	Craft and related trade workers	66 (6.2)	46.23±12.40
	Equipment, machine operation, and assembly workers	4 (0.4)	46.23±15.10
	Elementary workers	28 (2.6)	47.80±10.15
Total		1,072 (100)	46.63±12.86

**Table 2.** Health-related productivity loss and its 95% confidence intervals according to tertile of occupational stress levels (unit: percent points)

Occupational stress^1^	Model 1	Model 2	Model 3
Low	Reference	Reference	Reference
Intermediate	20.41 (16.01, 24.81)	20.41 (16.01, 24.81)	19.19 (13.23, 25.13)
High	23.41 (19.08, 27.74)	23.29 (18.96, 27.62)	21.17 (15.30, 27.04)

Model 1 was crude; Model 2 was adjusted for gender and age; Model 3 was adjusted for gender, age, education level, household income, occupation, and underlying medical conditions.

1 Estimated by a generalised linear model in contrast to the reference group (low organisational stress).

**Table 3.** Health-related productivity loss and its 95% confidence intervals according to tertiles of each category of occupational stress level (unit: percent points)

Variables	Occupational stress^1^		
	Low	Intermediate	High
Job demand (Q1)	Reference	14.21 (8.13, 20.28)	19.14 (13.07, 25.22)
Insufficient job control (Q2)	Reference	0.01 (-6.49, 6.50)	-0.57 (-6.93, 5.80)
Interpersonal conflict (Q3)	Reference	-2.51 (-10.07, 5.04)	7.00 (-0.77, 14.78)
Job insecurity (Q4)	Reference	7.88 (0.97, 14.79)	16.82 (10.94, 27.71)
Organisational system (Q5)	Reference	3.93 (-2.45, 10.33)	8.79 (2.70, 14.88)
Lack of reward (Q6)	Reference	2.22 (-4.84, 9.36)	6.95 (1.23, 1.27)
Occupational climate (Q7)	Reference	10.44 (4.11, 16.77)	21.56 (15.50, 27.61)

The model was adjusted for gender, age, education level, household income, occupation, and underlying medical conditions.

1 Estimated by the generalised linear model and contrast to the reference group (low occupational stress).
